# Designing a new orthopaedic trauma meeting proforma to improve documentation in a large UK teaching hospital

**DOI:** 10.1308/rcsann.2025.0043

**Published:** 2025-07-24

**Authors:** DJ Warrington, CR Yip, A Kassir, N Nadar, K Elcock, C Peach

**Affiliations:** ^1^ Manchester Foundation NHS Trust, UK; ^2^ The University of Manchester, UK

**Keywords:** Orthopaedic, Documentation, Quality improvement, Trauma meeting

## Abstract

**Introduction:**

In England, around two million fractures occur annually, with 250,000 requiring hospital admission. At Wythenshawe Hospital, the Trauma and Orthopaedic service discusses 10–15 new patient cases every weekday. We aimed to design and implement a structured proforma for the trauma meeting to ensure clear documentation of trauma meeting discussions and orthopaedic plans for each new patient at Wythenshawe Hospital.

**Methods:**

Based on a literature search, input from orthopaedic surgeons and analysis of existing documentation, we created a proforma. We collected data in four phases: pre-implementation (1–10 October 2022), post-initial proforma (11–20 October 2022), post-updated proforma (20–30 October 2022) and long-term effectiveness (20–24 November 2023).

**Results:**

Phase 1: 90 cases reviewed; 64% had inadequate documentation. Key details were often missing. Phase 2: After proforma implementation, 98 cases reviewed; documentation increased to 94%. Significant improvements in recording consultant names (92%), imaging (59%) and diagnosis (80%). Phase 3: After feedback update, 108 cases reviewed; 88% had documentation. Improvements in documentation of imaging (85%) and weight-bearing status (57%). Phase 4: One year later, 85 cases reviewed; documentation at 84%. Key details such as consultant names and imaging reached 100% completion, diagnosis at 97%.

**Conclusions:**

This study proposes a standardised trauma meeting proforma to enhance the efficiency and accuracy of trauma meeting documentation. Our findings highlight the need for professional bodies to establish guidelines for trauma meeting handovers. We encourage further research into effective trauma meetings and suggest our proforma as a template for other orthopaedic departments to adapt to their needs.

## Introduction

There are around two million fractures per year in England, with around 250,000 fracture admissions per year.^1^ The most common types of fractures requiring admission are hip fractures, distal radius fractures, ankle fractures and hand fractures.^2^ Wythenshawe Hospital is an acute teaching hospital in the Manchester University NHS Foundation Trust (MFT). We have a large Trauma and Orthopaedic Department consisting of 15 consultant surgeons, with around 50–70 orthopaedic inpatients on an average day.

A trauma meeting is a crucial multidisciplinary meeting of healthcare professionals involved in the care of trauma and orthopaedic patients. The discussions in trauma meetings focus on critical aspects of trauma care, including patient assessments, diagnostic findings, conservative versus surgical interventions and postoperative management. This interdisciplinary setting facilitates the exchange of knowledge, expertise and perspectives, contributing to improved decision-making and enhanced patient outcomes.

Trauma meetings also play a crucial role in planning the trauma theatre list, facilitating handover from the night team to the day team, and are considered a central element of the orthopaedic life in the hospital.^3^ During these meetings, healthcare professionals from various specialties, including orthopaedic surgeons, orthopaedic resident doctors, physicians, physiotherapists, theatre staff, anaesthetists, trauma nurses, scrub nurses, medical students and other relevant personnel of the trauma multidisciplinary team (MDT) come together to review and discuss admitted trauma cases and new inpatient referrals to orthopaedics. Members of the MDT who are not present at the trauma meeting, such as other doctors, occupational therapists, pharmacists, ward nurses and other healthcare staff, rely on accurate documentation of the trauma meeting for communication. The trauma meeting documentation acts as a form of handover of information. Clear documentation ensures that the team can complete their role in the patient journey safely and effectively.

Accurate and comprehensive record keeping in healthcare is crucial due to its impact on patient care, safety and the overall quality of healthcare services. Regulatory bodies like the General Medical Council, the Royal College of Surgeons of England and the British Medical Association emphasise the importance of record keeping for several reasons.^4–8^ First, it ensures continuity of care by providing essential information about a patient’s medical history, diagnoses, treatments and outcomes, enabling informed decision-making. Second, it facilitates effective communication and collaboration among healthcare professionals, promoting cohesive teamwork and consistent care. Third, accurate records reduce the risk of medical errors, ensuring patient safety. Fourth, maintaining accurate records is a legal and ethical requirement for healthcare professionals. Finally, records serve as a valuable resource for quality improvement initiatives, allowing for evaluation, identification of areas for enhancement and monitoring patient outcomes.

MFT recently implemented an electronic patient record (EPR) system called HIVE in September 2022.^9^ All patient notes and results are on this system and are available instantly to all staff involved in a patient’s care. This means that it is possible to write a trauma meeting note that is instantly accessible to all members of the MDT. However, without a standardised proforma, foreseeable issues remained unaddressed. A recognisable format would allow for these issues to be addressed with the senior doctors present to reduce delays in decision making. Additionally, it would ensure the quality of documentation was standardised.

Good quality of handover results in better patient care and is encouraged by the BMA.^10^ Research has shown that members of MDT meetings report clear documentation of the meeting to be important.^11^ Furthermore, clinicians felt that documenting in electronic form allowed for easy access for recall of events.^12^ There are no UK guidelines on how trauma meetings should be organised, or guidelines on how trauma meetings should be documented.

We therefore set out to create our own standardised trauma meeting proforma to improve the consistency and accuracy of trauma meeting documentation. We designed this standardised trauma meeting proforma through collaboration with the MDT as well as reviews and audits of its effectiveness. The aim of this project was to ensure consistent and clear documentation of trauma meeting MDT discussions for each patient discussed in the daily trauma meeting at Wythenshawe Hospital.

## Methods

### Literature search

We performed a literature search for papers relating to trauma meeting documentation on MEDLINE and EMBASE from inception to 27 January 2023. A copy of the literature search is available in Figure S2. We used the following search strategy:

1. ((handover* or meeting* or proforma) adj1 (orthop?edic* or trauma)).ti,ab.
2. (guideline* or audit*).ti,ab.
3. 1 and 2

### Creation of trauma meeting proforma

**Figure 1:**
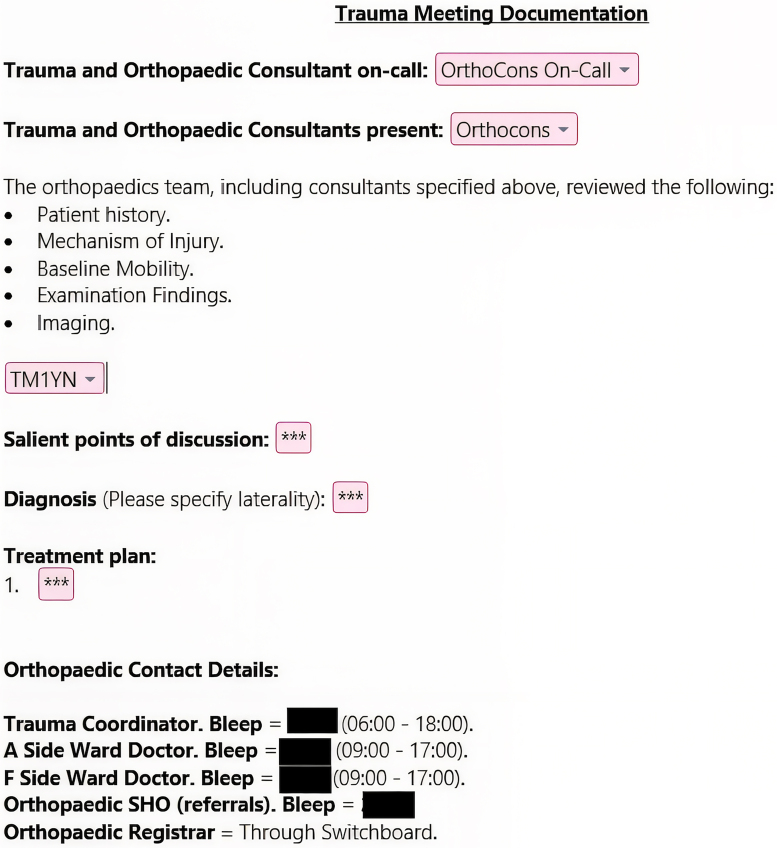
Trauma meeting documentation proforma

We used a combination of the results in our literature search, opinions of orthopaedic surgeons in our department and an analysis of previous documentation to create an initial trauma meeting proforma. Our EPR system allows for the creation of shortcuts and so we were able to design the note to quickly bring up our proforma if a doctor types in “.T1” into a note. After the implementation of the initial trauma meeting proforma, we sought feedback from the wider MDT including: orthogeriatricians, pharmacists, physiotherapists, occupational therapists, nurses, trauma coordinators and orthopaedic resident doctors. This resulted in a final version of the trauma meeting proforma (Figure 1).

### Data collection

We retrospectively reviewed all patients discussed in the trauma meeting at Wythenshawe Hospital in four phases. The first phase of data collection was to establish a baseline of trauma meeting documentation pre-implementation of a proforma; these data were collected between 1 and 10 October 2022. The second phase of data collection was postimplementation of the initial trauma meeting proforma, with data collected between 11 and 20 October 2022. The third phase of data collection was following the implementation of an updated trauma meeting proforma based on multidisciplinary feedback and data were collected between 20 and 30 October 2022.

The fourth and final phase of data collection was one year later to review the updated trauma meetings proforma’s long-term effectiveness. These data were collected between 20 and 24 November 2023. Prior to this data collection, we produced a short video and a poster as a guide for new doctors on the standards expected from a trauma meeting discussion and documentation (Figure S3).

## Results

### Phase 1 – Prior to implementation of a proforma

**Figure 2:**
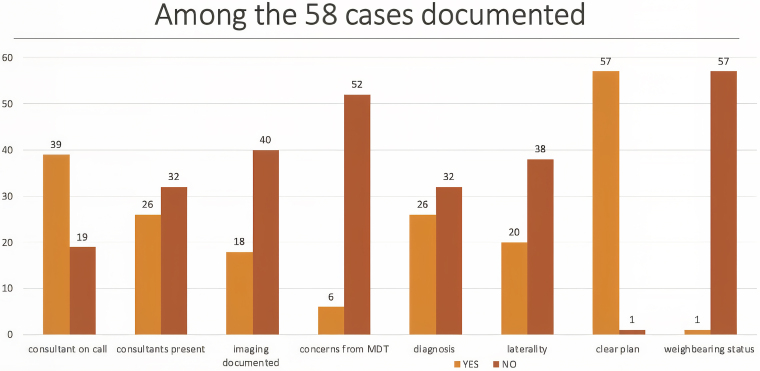
Trauma meeting documentation standards prior to implementation of a proforma

The first round of data collection was conducted from 1 to 10 October 2022, at Wythenshawe Hospital, predating the implementation of the proforma. During this period, a total of 90 cases were reviewed. Of these, 58 cases (64%) had some form of documentation, while 32 cases (36%) lacked any documentation. Specifically, 43% of the cases included documentation of the name of the consultant on call, 29% documented the names of other consultants present, 20% recorded the type of imaging utilised, 7% documented concerns raised during the MDT discussion, 29% provided documentation of the diagnosis, 22% included documentation of laterality, 63% outlined a clear plan and 1% specified weightbearing status. These findings are represented in Figure 2.

### Phase 2 – After implementation of a proforma

**Figure 3:**
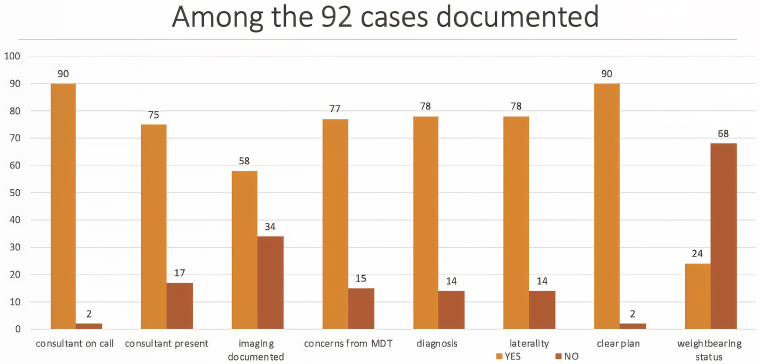
Trauma meeting documentation standards after the implementation of a proforma

The second round of data collection was performed between 11 and 20 October 2022 at Wythenshawe Hospital, following the implementation of the proforma. A total of 98 cases were discussed in this study period; 92 cases (94%) had documentation of the trauma meeting discussion and 6 cases (6%) had no documentation. This was a 47% increase of cases discussed having some form of documentation after the implementation of the proforma. Of the 98 cases, 92% had documentation of the name of the consultant on call (114% increase after implementation of proforma), 77% had documentation of the names of other consultants present in the trauma meeting (166% increase), 59% had documentation of the imaging discussed (195% increase), 79% had documentation of concerns from the MDT discussion (1,029% increase), 80% had clear documentation of the diagnosis (175% increase), 80% had documentation of the laterality (264% increase), 92% had a clear plan documented (46% increase) and 24% had documentation of the weightbearing status (2,300% increase). These findings are represented in Figure 3.

### Multidisciplinary survey

Multidisciplinary feedback was obtained from the staff at Wythenshawe Hospital to assess the functionality of the proforma and identify areas for improvement after the second round of data collection. We interviewed three orthogeriatricians, five physiotherapist/occupational therapists, two pharmacists, one ward nurse, one trauma coordinator and three orthopaedic surgeons. The survey results showed that different health care professionals had different priorities when assessing the trauma meeting documentation (Figure S4).

The orthogeriatricians wanted the following information: decision for surgical intervention, planned surgical procedure and the timing of the surgery (morning or afternoon). For frail patients with multiple comorbidities, they wanted resuscitation discussions to be clearly documented.

The physiotherapists/occupational therapists wanted the following information: type of procedure performed, weightbearing status, diagnosis including laterality and relevant comorbidities that could impact a patient’s recovery following surgery.

Pharmacists wanted the following information: the decision for surgical intervention and weightbearing status to guide fasting instructions and venous thromboembolic prophylaxis.

Nursing staff wanted the following information: the timing of the surgery including the fasting instructions, and whether the patient was aware of the management plan.

Trauma coordinators wanted the following information: the specific actions needed prior to surgery. This included completion of essential blood tests and electrocardiogram, documentation of resuscitation status and consent form with the correct procedure, and laterality.

Orthopaedic consultants wanted the following information: the completion of essential blood tests, documentation of resuscitation status and consent form. In addition to that, focused medical and drug history, and examination findings should be documented.

### Phase 3 – After implementation of revised proforma

The third round of data collection was performed between 20 and 30 October 2022 at Wythenshawe Hospital following further changes to the proforma based on multidisciplinary feedback. An additional standard was added to the proforma for patients who require surgery titled ‘Theatre Checklist’.

A total of 108 cases were discussed during this study period; 95 cases (88%) had documentation of the trauma meeting and 13 cases (12%) had no documentation. This is a slight decrease in the total number of trauma meetings documented compared with phase 2. Of these 108 cases, 88% had documentation of the name of the consultant on call (4% decrease compared with phase 2), 88% had other consultants present documented (14% increase), 85% had imaging discussed documented (44% increase), 65% had concerns from the MDT documented (18% decrease), 69% had documentation of clear diagnosis (14% decrease), 71% had documentation of laterality (11% decrease), 86% had clear plan documented (7% decrease) and 57% had weightbearing status documented (138% increase).

**Figure 4:**
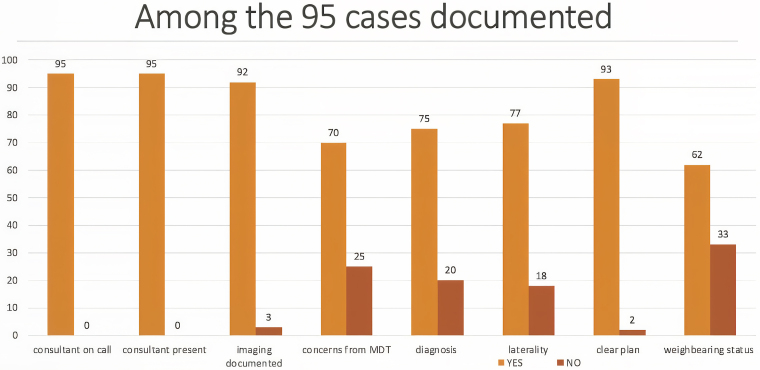
Trauma meeting documentation standards postimplementation of proforma with changes based on multidisciplinary feedback after round 2

Among the 108 cases, 57 cases required surgical intervention. Only 6 cases (11%) had completed the newly implemented theatre checklist, and the remaining 51 cases did not complete the theatre checklist. These findings are represented in Figure 4.

### Phase 4 – One year after implementation of proforma

A re-audit of the effectiveness of the new trauma meeting proforma was conducted between 20 and 24 November, 2023 at Wythenshawe Hospital. During this period, a total of 85 cases were reviewed; 71 cases (84%) had some form of documentation, which is a 31% increase from phase 1.

Specifically, 100% of the cases included documentation of the name of the consultant on call, the names of the other consultants present and imaging discussed. There were no concerns raised during the MDT discussion documented; however, this section was removed from the proforma. Of these 85 cases, 97% provided documentation of the diagnosis, 75% included documentation of laterality, 98% documented a clear plan and one note discussed weightbearing status as this section was removed from the proforma.

## Discussion

This quality improvement project has demonstrated that by serial implementation of a structured proforma in an acute hospital setting, the quality and consistency of clinical documentation from MDT meeting discussions can be improved. Furthermore, by recognising the turnover of staff members in the department, the addition of accessible training materials has ensured that these improved results have been effective in the long term.

### Development of trauma meeting proforma

Prior to this project, trauma meeting discussions occurred without a standardised documentation system. There was often no record of the trauma meeting discussion in the patients’ notes and the system relied on resident doctors and trauma coordinators taking their own post hoc notes. Any concerns raised by other members of the MDT would require communication with a senior doctor who may be in theatres, clinics or conducting ward rounds. There are certain decisions that are unique to orthopaedic patients that are predictable and should be documented clearly early in the patient’s admission. These include information such as exact operation to be performed, or requirements to withhold certain medications preoperatively. This information is critical for the patient’s management and a lack of clear documentation can result in delays and errors in decision making and treatment of patients.^13^ This can result in staff confusion and compromise of patient safety and satisfaction.^14,15^

We believed that it was important to develop a standardised trauma meeting proforma as it is well known that standardising documentation in healthcare can (1) improve consistency, (2) improve completeness of documentation, (3) improve accuracy of documentation, (4) improve access to essential clinical information, and (5) support quality improvement and research.^16^ We encourage other orthopaedic departments to use our proforma as a template and to audit whether it improves the accuracy of documentation.

### Improvement of trauma meeting proforma

Our trauma meeting proforma has been active in Wythenshawe’s busy orthopaedic department every day for the last 18 months. As a result, it has been through 18 months of iterative testing with small, gradual updates based on formal and informal feedback in real time.

Although the trauma meeting is an important part of the day, it is also the first meeting of the day and most of its attendees have many demands on their time, including clinics, operating lists and educational demands. We found that when we added new sections to our proforma, such as a theatre checklist, users completed the core elements of the proforma (88% documentation rate), but mostly left the additional sections blank. We found that members of the MDT varied significantly in what important information they wanted from the trauma meeting documentation. Anecdotally, orthopaedic surgeons felt that clear documentation of the trauma meeting was essential for medico-legal purposes in an increasingly litigious working environment. They also raised concerns with the weightbearing status section of the proforma; often patients admitted overnight were awaiting consultant review and the orthopaedic surgeons felt this was required before the weightbearing status could be documented for some of the patients, hence its removal from the proforma.

### Improvement in trauma meeting documentation and resulting benefits

Our paper demonstrates that there is a clear improvement in the documentation of the trauma meeting with our standardised proforma. While documenting the consultants involved in the discussion, the diagnosis and the plan may seem like an obvious practice, it was not being done effectively before the introduction of our proforma.

Research demonstrates that there is an overall incidence of in-hospital adverse events of 9.2%, with a median percentage of preventability of 43.5%.^17^ In 2008, the World Health Organization (WHO) published the ‘WHO Surgical Safety Checklist’, which is well known by all UK surgeons as it was made mandatory in 2009.^18,19^ This checklist has been shown to reduce the rate of deaths and surgical complications by up to one-third.^19^ Atul Gawande also discusses the benefits of checklists in his book ‘The Checklist Manifesto’.^20^ Furthermore, the Royal College of Physicians has also called for standardised handover processes to improve patient outcomes.^21^ Although we cannot present any data to evidence that our trauma meeting proforma improves outcomes, we believe that the literature supporting checklists in surgery and importance of standardised handovers is compelling and supports the introduction of our trauma meeting proforma more widely.

### Limitations

While this study provides valuable insights into the development of a proforma to improve the documentation of the orthopaedic trauma meeting in a large UK teaching hospital, potential limitations should be acknowledged.

The first limitation is in relation to a lack of consistency of the users of the trauma meeting proforma. In the UK, resident doctors rotate hospitals every four to six months and so this is an unavoidable issue. We mitigated this by producing a teaching video explaining the importance of good trauma meeting documentation and a step-by-step tutorial on how to do this and the expected standards. This video is included in the induction pack of all new doctors at Wythenshawe’s orthopaedic department.

The second limitation is in relation to a lack of previous research or guidelines for trauma meetings. This paper calls for further research and evaluation of trauma meeting structures and proformas to evaluate their effectiveness.

## Conclusion

This paper proposes a new standardised trauma meeting proforma to facilitate efficient and accurate discussion and documentation of the trauma meeting. We believe that this proforma provides a new gold standard for trauma meeting documentation, balancing the requirements of accurate documentation with the time constraints of a busy orthopaedic department. Furthermore, we have shown that this proforma is effective at improving the documentation of the trauma meeting and has been adopted successfully in another UK hospital. We encourage orthopaedic departments to adapt our proforma to suit their local requirements.

This paper demonstrates that there is a need for professional bodies that regulate surgeons and physicians to draft professional guidance and audit standards for handovers such as the trauma meeting. This paper also encourages further research into effective trauma meetings, including the evaluation of the potential use of artificial intelligence to help save time spent documenting and improve note-taking accuracy, and we encourage the use of our proforma as the basis for this.

## Data Availability

The data that support the findings of this study are available on request from the corresponding author.
